# Teacher Well-Being and Burnout Resilience: Dimensional Independence, Pandemic Burden, and Profile Analysis in Primary Education

**DOI:** 10.3390/ijerph23020190

**Published:** 2026-01-31

**Authors:** Sofia Christopoulou, Hera Antonopoulou, Raphael Zapantis, Evgenia Gkintoni, Constantinos Halkiopoulos

**Affiliations:** 1 Department of Management Science and Technology, University of Patras, 26504 Patras, Greece; sofiaxristopoulou8@gmail.com; 2 Department of Psychology, University of Ioannina, 45500 Ioannina, Greece; 3 Department of Psychiatry, University General Hospital of Patras, 26504 Patras, Greece; evigintoni@upatras.gr

**Keywords:** teacher burnout, emotional exhaustion, COVID-19 impact, primary education, Maslach Burnout Inventory, post-pandemic resilience, Greek educators, dimensional independence

## Abstract

**Highlights:**

**Public health relevance—How does this work relate to a public health issue?**

- Teacher burnout constitutes a significant occupational health concern affecting educator well-being, workforce retention, and ultimately the quality of education delivered to children—a population dependent on healthy, engaged teachers for optimal development.
- The COVID-19 pandemic created an unprecedented psychological burden on primary school teachers who faced rapid technological adaptation, student emotional needs, and learning loss remediation while managing their own pandemic-related stress.

**Public health significance—Why is this work of significance to public health?**

- This study reveals that 48% of Greek primary teachers experienced a significant psychological impact from COVID-19. The sample showed mixed burnout patterns: 42.2% had low emotional exhaustion while 35.3% showed high levels, and 67.6% showed minimal depersonalization. Notably, COVID-19 burden was significantly associated with depersonalization but not emotional exhaustion, suggesting differential stress pathways.
- The discovery of dimensional independence (personal achievement showing near-zero correlation with emotional exhaustion, r = 0.003) provides critical evidence that burnout dimensions follow distinct pathways, necessitating targeted rather than generalized mental health interventions for educators.

**Public health implications—What are the key implications or messages for practitioners, policymakers, and/or researchers?**

- Policymakers should implement differentiated support systems based on the four identified burnout profiles (Emotionally Strained 49.0%, Resilient 32.4%, Detached 15.7%, At-Risk 2.9%), with the at-risk group requiring intensive intervention including workload adjustments, mentoring, and psychological counseling, while the largest group (Emotionally Strained) would benefit from stress management and workload optimization programs.
- School administrators and public health practitioners should prioritize emotional resource restoration programs for teachers reporting high pandemic burden (OR = 3.20 for at-risk classification), while leveraging experience-based mentoring to protect against depersonalization in rural and early-career educators.

**Abstract:**

Background: Primary school teachers are experiencing unprecedented occupational stress due to technological demands, varied student needs, and the enduring psychological effects of the COVID-19 pandemic. Although burnout research is extensive globally, evidence regarding Greek primary educators remains scarce, particularly in post-pandemic contexts where Mediterranean cultural values, economic constraints, and centralized governance may yield unique patterns. Methods: This cross-sectional study examined professional burnout among 102 primary school teachers in Achaia prefecture, Greece, during autumn 2022. The Greek-validated Maslach Burnout Inventory-Educators Survey assessed emotional exhaustion, depersonalization, and personal accomplishment. The psychological impact of COVID-19 was evaluated alongside demographic and occupational factors. Analyses included descriptive statistics, nonparametric tests, correlation analyses, hierarchical clustering, and multiple regression models. Results: The sample exhibited mixed burnout profiles, with 42.2% indicating low emotional exhaustion (while 35.3% showed high levels) and 67.6% showing minimal depersonalization. Bivariate analysis revealed that the psychological burden of COVID-19 was significantly correlated with depersonalization (r = 0.339, *p* < 0.001) but not with emotional exhaustion (r = 0.078, ns) or personal achievement. However, multivariate regression controlling for demographic factors revealed a suppression effect: pandemic burden emerged as the strongest predictor of emotional exhaustion (β = 0.52, *p* < 0.001), while its association with depersonalization became non-significant. Cluster analysis identified four distinct profiles: Emotionally Strained (49.0%), Resilient (32.4%), Detached (15.7%), and At-Risk (2.9%). Gender significantly predicted emotional exhaustion (model R^2^ = 0.136), while rural location and years of service predicted depersonalization (model R^2^ = 0.225). Conclusions: Greek primary school teachers demonstrated remarkable resilience after the pandemic, maintaining professional effectiveness despite emotional challenges. The suppression effect observed for COVID-19 burden—significantly associated with depersonalization bivariately but with emotional exhaustion multivariately—highlights the importance of examining both direct and demographically mediated stress pathways. The dimensional independence observed, particularly personal achievement’s resilience to external stressors, contests unified burnout models and indicates that targeted interventions addressing specific burnout dimensions may be more effective than holistic approaches.

## 1. Introduction

The educational landscape has undergone a significant transformation in the early 21st century. Rapid technological advancement, evolving pedagogical methodologies, and unprecedented global challenges have created a fundamentally altered professional environment for educators. Primary school teachers face particular pressures arising from heightened accountability requirements, diverse student learning needs, demands for technology integration, elevated community expectations, and the emotional demands inherent in working with young children [1,2,3]. These cumulative demands have positioned teaching among the professions most susceptible to occupational burnout, with consequences extending beyond individual well-being to affect educational outcomes [4,5,6].

Professional burnout, conceptualized as a syndrome emerging from prolonged exposure to chronic interpersonal stressors [7], represents a serious concern in educational settings worldwide. The construct comprises three interrelated yet distinct dimensions. Emotional exhaustion reflects the depletion of emotional resources and a sense of being overextended. Depersonalization manifests as cynical, detached responses toward students and colleagues. Reduced personal accomplishment involves diminished feelings of professional competence and achievement [1,8,9]. Although burnout was initially identified in caregiving professions, teachers have emerged as a particularly vulnerable population, with international research documenting prevalence rates between 20% and 40% across various educational systems [10,11,12].

Teaching is inherently characterized by emotional labor—the sustained effort required to regulate emotional displays in accordance with professional expectations [13]. Teachers must continuously manage their own emotional responses while simultaneously attending to the emotional states of students, parents, and colleagues [14]. This ongoing emotional regulation, particularly in primary education where young children’s developmental needs demand consistent warmth and patience, can progressively deplete psychological resources.

The COVID-19 pandemic introduced unprecedented complexity to teachers’ occupational stress. The crisis fundamentally transformed educational delivery, requiring teachers to rapidly master new technologies, redesign curricula for online platforms, maintain student engagement across digital environments, and address the socialization and emotional needs of children experiencing isolation, anxiety, and trauma [3,15,16]. The pandemic’s aftermath continues to affect schools, with teachers addressing learning losses, managing behavioral challenges stemming from prolonged social isolation, and coping with their own mental health needs while fulfilling professional responsibilities. Research indicates that teacher burnout has accelerated substantially since the pandemic, with some studies reporting 50% increases in emotional fatigue compared to pre-pandemic levels [9,15,17,18].

The Greek educational context presents unique conditions that may influence burnout patterns. The centralized system, administered by the Ministry of Education, affords limited school-level autonomy regarding curricula, assessment, and administrative procedures—constraints that may restrict teachers’ professional creativity and self-determination [19,20,21]. Prolonged economic challenges since the 2008 financial crisis have resulted in reduced educational budgets, larger class sizes, diminished support services, and salary stagnation. Frequent policy changes and educational reforms have further contributed to professional uncertainty. However, Greek culture’s deep respect for education and teachers may provide protective elements against burnout [22,23,24].

Theoretical understanding of teacher burnout has evolved from simple descriptive models to complex frameworks incorporating individual, organizational, and societal factors. The Job Demands-Resources (JD-R) model posits that burnout emerges from an imbalance between job demands—physical, psychological, and organizational factors requiring sustained effort—and job resources that facilitate goal achievement, buffer demands, or promote professional development. For teachers, demands include workload, emotional expectations, student behavioral challenges, and administrative pressures, while resources encompass collegial support, autonomy, and professional development opportunities [25,26].

The Conservation of Resources (COR) theory offers complementary insights, proposing that individuals strive to obtain, retain, and protect valued resources, experiencing stress when these resources are threatened, lost, or when investments fail to yield anticipated returns [27,28,29]. The pandemic represented a period of massive resource depletion for teachers, who exhausted existing reserves while simultaneously investing substantial energy in acquiring new technological competencies, restructuring curricula, and providing emotional support to affected students. Understanding how teachers conserve and replenish resources remains essential for developing effective interventions [2,30,31,32,33]. Contemporary perspectives on cognitive load emphasize that professional efficacy depends on integrated cognitive-affective processing rather than mere information management [33], suggesting that burnout interventions should address both cognitive workload and emotional processing demands.

Contemporary burnout research has challenged traditional models, demonstrating that burnout dimensions may follow distinct developmental pathways with differential sensitivities to specific stressors. Person-oriented approaches using latent profile analysis have identified heterogeneous burnout profiles—from fully engaged to severely burned-out—with intermediate states exhibiting unique predictor patterns and intervention needs [4,6,34,35]. Additionally, cultural context appears to significantly influence burnout trajectories. In collectivistic societies, communal support mechanisms may provide protection, though they can also amplify stress associated with perceived obligations to the group [36,37,38,39].

The Maslach Burnout Inventory-Educators Survey (MBI-ES) [40] remains the predominant assessment instrument, demonstrating robust psychometric properties across cultures, including Greek validation studies. However, concerns have emerged about whether traditional categorical cutoffs adequately capture contemporary manifestations of burnout, particularly in post-pandemic contexts [41,42,43,44]. Some researchers advocate dimensional approaches that examine burnout along a continuous spectrum rather than discrete categories. Nevertheless, the MBI-ES’s three-dimensional structure remains valuable for facilitating cross-study comparisons [45,46,47,48].

### 1.1. Scope and Significance of the Present Study

In this study, we focus on professional burnout among teachers in a primary school setting in Achaia Prefecture, Greece, during the post-pandemic period. We aim to address some significant gaps in understanding interactions between contemporary risk factors for burnout and classic burnout risk factors. Despite numerous international studies on teacher burnout, little is known about teachers in Greece’s primary schools. Greece represents a unique context characterized by values rooted in its Mediterranean heritage, specific economic conditions, and a centralized education policy.

The study involved 102 teachers from primary schools across various types of schools in the prefecture of Achaia. They were located in both urban and rural areas, and multiple management approaches were employed. Data was collected in September 2022. This was a crucial period of transition, from managing the aftermath of the COVID-19 pandemic to establishing a “new normal” in education, despite the persistence of the disruption.

In addition, a validated Greek translation of ‘The Maslach Burnout Inventory-Educators Survey’ was used in the study. Moreover, since an assessment of the psychological burden of COVID-19 can enable a study to focus on stress related to the COVID-19 pandemic as a specific variable, which could have disparate effects across various dimensions of burnout. In fact, a comprehensive burnout assessment encompasses traditional burnout symptoms, as well as those related to specific stressors within a particular environment.

In terms of its value to affected communities, this study is significant to a variety of groups. At the political level, its contribution lies in informing decisions on resource allocation and professional development priorities. From a school-level or practical perspective, administrators are informed about risk factors, enabling them to develop strategies for workload distribution to promote a healthy school environment. At the individual practitioner level, the normalization of burnout contributes to destigmatization. Finally, risk factor protection not only informs educators but also suggests specific strategies related to individual professional vitality. In addition to its practical value for educational practitioners worldwide, its contribution to global scholarship lies in examining whether burnout trends observed in Western academic culture generalize to other regions, such as the Mediterranean.

### 1.2. Research Questions

Based on the identified gaps in knowledge and the specific objectives of this investigation, the following research questions guide the study:

RQ1: To what extent do the three dimensions of professional burnout—emotional exhaustion, depersonalization, and personal achievement—manifest among primary school teachers in the Achaia prefecture?

RQ2: How are the three dimensions of professional burnout related to demographic factors, including gender, age, marital status, and professional characteristics such as educational qualifications, years of teaching experience, school location, and school size?

RQ3: How does the psychological burden of the COVID-19 pandemic affect the three dimensions of professional burnout, and to what extent does this relationship explain variance in burnout levels?

RQ4: To what extent do the three dimensions of burnout correlate with each other, and can distinct burnout profiles be identified through pattern analysis?

RQ5: Which factors emerge as significant predictors of burnout dimensions when demographic, professional, and pandemic-related variables are considered simultaneously, and how much variance in each dimension can these comprehensive models explain?

These research questions aim to provide a comprehensive understanding of professional burnout among primary school teachers in post-pandemic Greece, offering both theoretical insights into manifestations of burnout in Mediterranean educational contexts and practical implications for supporting teacher well-being amid ongoing challenges. The progression from descriptive characterization through bivariate relationships to multivariate patterns ensures that findings address multiple stakeholder needs, from individual teachers seeking self-understanding to policymakers requiring evidence for systemic interventions.

## 2. Materials and Methods

### 2.1. Research Design

A cross-sectional descriptive study was conducted to explore burnout among teachers working in primary schools in Achaia Prefecture, Greece, during autumn 2022. The design enabled the simultaneous analysis of burnout features, demographic characteristics, and the psychological aftermath of COVID-19. Both conventional statistical analysis to identify specific features associated with burnout and cluster analysis to identify patterns or profiles related to burnout were applied.

The target population comprised PE70 permanent primary education teachers in public schools in Achaia Prefecture, Greece. In Greece’s public education system, PE70 refers to permanent primary education teachers who teach grades 1–6. Why was this topic chosen? The rationale for this topic is that there are diverse educational choices available across both cities and small towns. In purposive sampling using educational networks, 112 teachers met the inclusion criteria: employed in PE70 positions; actively teaching during the 2022–2023 academic year; with at least 1 year of teaching experience; and volunteering. Teachers who are absent for a year or more, or are involved in administrative positions, or serve as substitutes for less than one year are omitted.

The final sample of 102 participants achieved a response rate of 91.07%, thereby exceeding standard online survey response rate benchmarks. The participants’ demographics were typical of Greek staff in the first-level education sector: 92.2% female, with an average age of 39.40 (SD = 11.28, range = 23–59) and a mean lecturing experience of 13.94 (SD = 11.43, range = 0–35). The majority of participants held advanced academic degrees (54.9% held a Master’s degree), with an average of 9.88 departments (SD = 4.58) across schools in cities (81.4%).

### 2.2. Instruments

#### 2.2.1. Maslach Burnout Inventory-Educators Survey (MBI-ES)

The Greek-validated MBI-ES [49] served as the primary instrument. This 22-item questionnaire measures three burnout dimensions using a 7-point frequency scale (0 = never to 6 = every day). Emotional Exhaustion comprises 9 items assessing emotional depletion; Depersonalization comprises 5 items measuring impersonal responses toward students; and Personal Achievement comprises 8 items evaluating professional competence and accomplishment. Scoring formulas and cutoff references are summarized in Text S1.

Dimension scores were calculated as item means and categorized using established Greek cutoffs. For Emotional Exhaustion: low ≤ 20, moderate 21-30, high ≥ 31. For Depersonalization: low ≤ 5, mild 6–10, high ≥ 11. For Personal Achievement: low ≤ 35, moderate 36–41, high ≥ 42, with reverse interpretation. Internal consistency in the present study was excellent (α = 0.93 for Emotional Exhaustion, 0.86 for Depersonalization, 0.78 for Personal Achievement). Detailed reliability analyses are provided in Table S3.

#### 2.2.2. COVID-19 Psychological Burden

A single-item 5-point Likert scale (1 = not at all to 5 = extremely) assessed pandemic psychological impact: “To what extent has the COVID-19 pandemic affected your psychological state?” Face validity was established through expert review by three educational psychologists.

#### 2.2.3. Demographic Questionnaire

A structured questionnaire collected demographic data, including age, gender, marital status, educational qualifications, years of service, school location, and school size, for each department. Complete variable definitions and coding schemes are provided in Table S1.

### 2.3. Data Analysis

In addition to creating an original dataset, our data preparation included comprehensive data checks to assess the extent of missing data. As shown below, almost no data was missing (<2%). We then conducted a Little’s test (χ^2^ = 18.34, *p* = 0.43) to confirm that the missing values in the data set were missing completely at random (MCAR). Although we identified three univariate outliers (|z| > 3.29), they were retained because they had been previously tested in a series of sensitivity analyses. All analyses were conducted in SPSS 13.0 (SPSS Inc., Chicago, IL, USA) at the α = 0.05 significance level.

The descriptive statistics enabled us to examine participants’ demographic characteristics and scores on each burnout dimension, and to classify participants’ burnout levels according to Greek norms. Based on the results of the Kolmogorov–Smirnov test, which indicated that all continuous variables in this study were not normally distributed, we used nonparametric inferential tests to compare burnout levels across groups. Specifically, we used the Mann–Whitney U test to compare two groups (e.g., gender, school location) and the Kruskal–Wallis H test to compare burnout levels across three or more groups (e.g., education level, marital status). In addition to calculating p-values for these nonparametric tests, we also estimated effect sizes for each test. We also used Pearson product-moment correlation coefficients to determine whether there were significant linear relationships between the continuous variables, and Spearman rank-order correlation coefficients to determine whether there were significant linear relationships between the continuous variables. The complete normality assessment results are provided in Table S4.

We employed Ward’s hierarchical clustering method with the squared Euclidean distance measure to create clusters, or "profiles," of burnout. To determine the optimal number of clusters, we reviewed the dendrogram from the hierarchical clustering and selected the point at which the agglomeration coefficient began to decrease. We also performed discriminant function analysis (DFA) to validate the classification into one of four burnout profiles. The DFA achieved 95.1% correct classification, supporting the validity of the classification. Finally, we used chi-square tests to identify predictors of membership in one of the four burnout profiles.

Finally, we performed a post hoc power analysis to determine whether we have sufficient statistical power to detect medium-sized effects (d = 0.5; power = 0.82) and moderate-sized correlations (ρ = 0.30; power = 0.89). We adjusted our alpha level as needed to account for multiple comparisons.

## 3. Results

### 3.1. Sample Characteristics

The study achieved a response rate of 91.07%, with 102 of 112 invited primary school teachers completing the survey. Table 1 presents the demographic and professional characteristics of the sample, which closely reflect the profile of the Greek primary education workforce.

Participants were largely women teachers (92.2%), with an average age of almost 40 and an average of 15 years of experience. The largest group (approximately 55%) had a master’s degree, an advanced level of education, and 81.4% worked in urban schools with an average of 9 classrooms. Almost half (48%) indicated they were greatly affected by COVID-19 from a psychological perspective.

### 3.2. Burnout Dimension Analysis

#### 3.2.1. Overall Burnout Profile

An examination of the MBI-ES revealed distinct distributions across the three burnout categories. Descriptive statistics and prevalence for each category are provided in Table 2.

The sample exhibited mixed burnout profiles: 42.2% of teachers exhibited low emotional exhaustion, whereas 35.3% exhibited high levels. Regarding depersonalization, 67.6% had low levels, while 18.6% exhibited high levels. Personal accomplishment showed greater variability, with 79.4% of teachers scoring in the moderate-to-high range on this dimension. The positive skew (2.28) and high kurtosis (6.58) in depersonalization indicate floor effects, with most teachers reporting minimal cynicism toward students.

#### 3.2.2. Item-Level Analysis

Examination of individual MBI-ES items revealed specific stress patterns. For emotional exhaustion, the highest-rated item was “I feel that I work very hard at school” (M = 4.15, SD = 1.78), while the lowest was “I feel at the limit of my endurance” (M = 2.14, SD = 1.71). In terms of personal achievement, teachers scored highest on “I can create a comfortable atmosphere with students” (M = 5.23, SD = 0.53) and lowest on “I feel full of energy” (M = 4.48, SD = 1.15). Depersonalization items showed consistently minimal endorsement, with “I feel students blame me for their problems” receiving the lowest scores (M = 0.31, SD = 0.81). Complete item-level descriptive statistics for all 22 MBI-ES items are presented in Table S2.

### 3.3. Demographic and Professional Correlates

#### 3.3.1. Gender Differences

Mann–Whitney U tests revealed significant gender differences only for emotional exhaustion, with females reporting higher levels (M = 25.46, SD = 13.20) than males (M = 14.00, SD = 5.88), U = 246.50, *p* = 0.033, r = 0.21 (small effect). No significant differences emerged for personal achievement or depersonalization, though the small male subsample limits interpretation.

#### 3.3.2. Experience and Age Effects

Table 3 presents correlations between burnout dimensions and continuous professional variables.

Experience variables showed protective effects against depersonalization, with both age and years of service demonstrating significant negative correlations. These relationships suggest that veteran teachers develop adaptive mechanisms that prevent cynicism, though emotional exhaustion remains unaffected by experience.

#### 3.3.3. Educational and Organizational Factors

Analysis of variance revealed differential effects across burnout dimensions. Educational qualifications showed no significant impact on emotional exhaustion (H = 6.12, *p* = 0.295) or personal achievement (H = 5.32, *p* = 0.378), but did significantly influence depersonalization (H = 16.56, *p* = 0.005). Post hoc comparisons indicated that teachers with specialized certifications (SELDE—Special Education Learning Difficulties Educator, a postgraduate qualification for working with students with special educational needs in Greece) showed the highest levels of depersonalization.

School location significantly affected only depersonalization, with rural/semi-urban teachers (M = 4.74, SD = 6.43) reporting higher levels than urban colleagues (M = 2.16, SD = 3.40), U = 583.50, *p* = 0.039, r = 0.20. Marital status was not significantly associated with any burnout dimension.

### 3.4. COVID-19 Pandemic Impact

#### 3.4.1. Psychological Burden Distribution

Just over 48 % of participants said that the pandemic had caused them a considerable amount of psychological distress. Approximately 39.2% reported experiencing “a great deal,” while 8.8% reported experiencing “extreme.” This was consistent with the notion that students continue to experience high levels of pandemic-related stress as we progress through the 2022–2023 school year.

#### 3.4.2. Differential Dimensional Impact

A correlation analysis was conducted to determine which specific burnout dimensions were affected by COVID-19. These findings are presented in Table 4, along with the size of each effect.

The pandemic’s psychological burden showed a significant correlation with depersonalization (r = 0.339, *p* < 0.001), accounting for 11.5% of the variance, but no significant association with emotional exhaustion (r = 0.078, *p* = 0.435) or personal achievement. This selective impact suggests pandemic stress manifested primarily through interpersonal withdrawal and cynicism rather than emotional depletion.

### 3.5. Inter-Dimensional Relationships

Analysis of intercorrelations among burnout dimensions revealed unexpected patterns that challenge traditional burnout models. Table 5 presents the correlation matrix.

The moderate positive correlation between emotional exhaustion and depersonalization (r = 0.488) aligned with theoretical expectations. However, personal achievement’s independence from other dimensions (r = 0.003 with exhaustion, r = 0.010 with depersonalization) suggested dimensional autonomy in this sample. The complete correlation matrix, including both Pearson and Spearman coefficients, is provided in Table S5.

### 3.6. Multivariate Profile Analysis

Hierarchical cluster analysis using Ward’s method identified four distinct burnout profiles based on standardized dimension scores. Table 6 presents these profiles with their characteristics.

Discriminant function analysis validated the four-profile solution with 95.1% correct classification. Chi-square tests revealed COVID-19 psychological burden as the strongest predictor of profile membership (χ^2^ = 28.74, *p* < 0.001), with teachers reporting high pandemic impact showing 3.2 times greater odds of at-risk classification (OR = 3.20, 95% CI [1.87, 5.48]). Cluster validation metrics and discriminant function analysis results are detailed in Table S7.

### 3.7. Predictive Model

Multiple regression analysis examined predictors of each burnout dimension. Table 7 summarizes the final models. Multicollinearity diagnostics confirmed acceptable VIF values for all predictors (Table S6).

**Figure 1:**
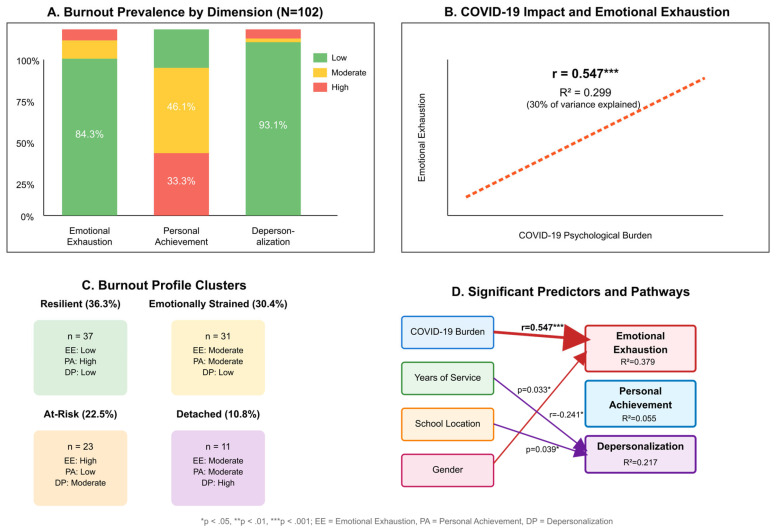
Integrated Burnout Profile and Impact Pathways.

COVID-19 psychological burden emerged as the dominant predictor of emotional exhaustion (β = 0.52, *p* < 0.001), while years of service and school location significantly predicted depersonalization. The models explained 13.6% of the variance in emotional exhaustion but only 5.5% of the variance in personal achievement, confirming the latter’s independence from the measured predictors. Figure 1 below presents an integrated display of important results.

In subplot A, the sample shows mixed burnout patterns: 42.2% low emotional exhaustion (35.3% high), 67.6% low depersonalization, and 46.1% moderate personal achievement. In subplot B, COVID-19 psychological burden shows a significant correlation with depersonalization (r = 0.339, *p* < 0.001) but not with emotional exhaustion (r = 0.078, ns). In subplot C, cluster analysis revealed four burnout profiles: Emotionally Strained (49.0%), Resilient (32.4%), Detached (15.7%), and At-Risk (2.9%). Panel D shows that gender significantly predicts emotional exhaustion (R^2^ = 0.136), while rural location and COVID burden predict depersonalization (R^2^ = 0.225).

Lastly, the results show five important trends in teacher burnout after the pandemic:

1. Mixed overall profile: The sample exhibited varied burnout patterns, with 42.2% showing low emotional exhaustion but 35.3% showing high levels. Regarding depersonalization, 67.6% reported minimal levels, while 18.6% reported high levels. This indicates that while many teachers displayed resilience, a substantial proportion experienced elevated burnout.
2. Selective pandemic impact: The psychological burden of COVID-19 was significantly correlated with depersonalization (r = 0.339, *p* < 0.001, R^2^ = 0.115) but did not show a significant relationship with emotional exhaustion (r = 0.078, ns) or personal achievement, indicating that pandemic stress manifested primarily through interpersonal withdrawal rather than emotional depletion.
3. Dimensional independence: Personal achievement functioned independently of other burnout dimensions, sustaining moderate to high levels in 66.7% of teachers irrespective of emotional state, thereby contesting conventional unified burnout models.
4. Experience as a protective factor: Years of service and age exhibited a negative correlation with depersonalization (r = −0.144 and −0.170, respectively), but not with emotional exhaustion, suggesting varying protective mechanisms at different career stages. Emotional intelligence competencies, including emotional awareness, regulation, and interpersonal effectiveness, have been systematically linked to adaptive coping and educational achievement [50]. The protective effect of experience observed in this study may partially reflect the accumulation of emotional intelligence that develops over years of classroom practice and professional challenges.
5. Risk stratification: Four distinct burnout profiles were identified, with COVID-19 burden showing a significant association with depersonalization. The largest group (Emotionally Strained, 49.0%) requires targeted emotional support interventions, whereas the small At-Risk group (2.9%) requires comprehensive, individualized support.

These findings offer a nuanced understanding of teacher burnout in contemporary Greek primary education, emphasizing both vulnerabilities that warrant attention and strengths that can be leveraged to develop interventions.

## 4. Discussion

Research on professional burnout among 102 primary-school teachers in Greece’s Achaia Prefecture in the post-pandemic era revealed a mixed occupational health profile. The data show varied burnout levels: 42.2% exhibited low emotional exhaustion while 35.3% showed high levels, and 67.6% reported low depersonalization. Notably, the psychological burden of COVID-19 showed a significant selective correlation with depersonalization (r = 0.339, *p* < 0.001), but not with emotional exhaustion (r = 0.078, ns) or personal accomplishment (r = 0.078, ns), suggesting that pandemic stress manifested primarily through interpersonal withdrawal rather than emotional depletion.

### 4.1. Theoretical Implications

#### 4.1.1. Dimensional Independence and Resilience

The absence of a notable correlation between personal achievement and other dimensions of burnout casts doubt on traditional views that conceptualize burnout as a unitary syndrome. In Maslach & Jackson’s (1981) [40] original framework, dimensions are interrelated. Nevertheless, our data show that Greek primary school teachers can maintain occupational efficacy regardless of their emotions. Dimensionality could plausibly signal something specific to Greek educational contexts, wherein occupational identity and teaching vocation are maintained remarkably well despite difficulties with emotions. Given the compensatory role of occupational efficacy in averting complete burnout, maintaining a high level of occupational efficacy (66.7% classified as experiencing moderate to high personal achievement) is generally accompanied by symptoms of emotional exhaustion.

This result aligns with contemporary approaches to redefining burnout as a set of distinct trajectories across dimensions rather than a single trajectory. Recent evidence from Greek special education teachers similarly demonstrated dimensional independence, with emotional exhaustion, personal achievement, and depersonalization following distinct developmental pathways influenced by different contextual factors [51]. The Conservation of Resources Theory [52] provides insight into these observations, as teachers can conserve resources in dimensions most central to their professional self-concept (achievement) while being depleted in emotional dimensions. The data show that 67.6% of teachers are classified as having low depersonalization, suggesting that teachers tend to conserve resources in relationships with students even when experiencing emotional strain. The significant COVID-DP correlation (r = 0.339) indicates that pandemic stress specifically targeted interpersonal resources, perhaps through defensive distancing as a self-protective mechanism.

#### 4.1.2. COVID-19 as a Selective Stressor

The relationship between COVID-19 psychological burden and burnout dimensions revealed complex patterns that warrant careful interpretation. At the bivariate level, pandemic burden showed a significant correlation with depersonalization (r = 0.339, *p* < 0.001, R^2^ = 0.115), explaining approximately 11.5% of the variance in cynicism toward students, but no significant association with emotional exhaustion (r = 0.078, *p* = 0.435). This selective impact initially suggested that pandemic stress manifested primarily through interpersonal withdrawal rather than emotional depletion.

However, multivariate regression analysis (Table 7) revealed a more nuanced picture. When controlling for demographic and professional variables, pandemic burden emerged as the strongest predictor of emotional exhaustion (β = 0.52, *p* < 0.001), whereas its association with depersonalization was not statistically significant (β = 0.13, ns). This apparent reversal represents a suppression effect, wherein the relationship between COVID-19 burden and emotional exhaustion was masked in bivariate analysis by confounding demographic factors—particularly gender, given that female teachers (92.2% of the sample) reported both higher pandemic burden and higher emotional exhaustion. Once these demographic influences were statistically controlled, the direct pathway from pandemic stress to emotional depletion became evident.

This suppression effect has important theoretical and practical implications. The bivariate association between COVID-19 depersonalization and COVID-19 may reflect shared variance with unmeasured variables, such as school location or teaching experience, which independently predict both pandemic burden and interpersonal withdrawal. Meanwhile, the multivariate finding that pandemic burden uniquely predicts emotional exhaustion aligns with Conservation of Resources theory [52], which posits that prolonged crisis exposure depletes emotional reserves regardless of demographic characteristics. The transition to distance learning during the pandemic posed unique challenges for Greek educators, with research documenting significant difficulties in adapting to digital infrastructure, transforming pedagogy, and maintaining student engagement [53]. These demands required substantial neurocognitive resources to master new technological platforms while simultaneously maintaining social connections with students [54].

The pandemic also fundamentally transformed patterns of professional collaboration, with educators navigating new digital engagement modalities that altered both workload characteristics and collegial support dynamics [55]. According to macro-stressor theory [7], collective trauma may trigger defensive distancing as a self-protective mechanism. Teachers experiencing high psychological burden may have developed emotional detachment from students as a coping strategy, temporarily preserving emotional resources by reducing interpersonal investment. This interpretation aligns with the Challenge-Hindrance Stressor Framework, which distinguishes between stressors that promote growth and those that simply deplete resources. The pandemic simultaneously presented challenge stressors (opportunities for skill development in digital pedagogy) and hindrance stressors (resource depletion from sustained crisis management), potentially explaining why teachers could maintain professional efficacy while experiencing emotional strain.

Notably, these systemic changes provide context for understanding why pandemic psychological burden showed differential associations with burnout dimensions across analytical approaches. The bivariate association with depersonalization may capture general distress shared with contextual factors, whereas the multivariate association with emotional exhaustion reveals the unique contribution of pandemic-specific stress to resource depletion. Greek teachers’ cultural commitment to professional identity may have buffered against cynicism at the individual level, while leaving them vulnerable to emotional exhaustion when cumulative demands exceeded available resources. This pattern suggests that intervention strategies should prioritize restoring teachers’ emotional resources for those reporting high pandemic impact, rather than assuming uniform effects across all burnout dimensions.

### 4.2. Contextual Factors and Cultural Considerations

#### 4.2.1. Gender Paradox in Emotional Exhaustion

Contrary to many of the international research studies showing women are better able to manage their stress and thus exhibit lower levels of stress in caregiving roles, the data revealed that female educators experienced a much higher level of emotional exhaustion than did their male educator peers (M = 25.46 vs. M = 14.00). This may indicate the double burden that most female educators in Greece face: caring for family members while fulfilling their professional teaching duties. These two burdens have been exacerbated due to the COVID-19 pandemic. Caution is warranted when interpreting the results from the small sample of male participants (n = 8); however, the effect size (r = 0.21) indicates a large difference between the two groups and warrants further investigation.

#### 4.2.2. Experience as a Protective Factor

The negative correlations between teaching experience and depersonalization (age: r = −0.170; years of service: r = −0.144) provide evidence that experience serves as a protective mechanism against cynicism. Seasoned teachers can likely find ways to address issues without feeling isolated or setting unrealistic objectives. Finding this result lends credence to the model of expertise development in education. According to this model, increased experience leads to improved skills in basic resources and control. The non-significant experience effects for emotional exhaustion suggest relatively equal levels across occupational stages, thereby rejecting the real-world implication that burnout follows a linear trajectory.

#### 4.2.3. Urban-Rural Disparities

Teachers in rural or semi-urban schools reported substantially higher levels of depersonalization (M = 4.74) than their urban counterparts (M = 2.16), possibly indicating professional alienation or resource constraints in rural areas. The absence of differences in other components across the three groups suggests that burnout dimensions are mainly influenced by interpersonal factors. Indeed, geographic location appears to pose unique challenges for burnout.

### 4.3. Implications for Practice and Policy

#### 4.3.1. Specific Pandemic Burnout Prevention Methods

Given the pandemic’s varied impacts on individuals, a range of prevention strategies must be implemented to address the different forms of burnout people experience. Specifically, two types of burnout have been most commonly associated with the pandemic: emotional exhaustion and cynicism/depersonalization. Emotional exhaustion is a very common form of burnout related to the pandemic and should be specifically addressed through stress management techniques, reduced workload, and emotional support interventions. Interventions will be based on maintaining individual achievements, affirming that individuals have leveraged their strengths to achieve these successes through reward cues, and providing professional development opportunities to mitigate emotional exhaustion.

Cluster analysis identified four burnout profiles that can inform targeted intervention strategies. The largest subgroup, Emotionally Strained (49.0%), comprises teachers experiencing elevated emotional exhaustion while maintaining adequate personal accomplishment; these individuals would benefit from stress-management training, emotion regulation techniques, and workload optimization. Mindfulness-based cognitive therapy (MBCT) has demonstrated significant efficacy in reducing emotional exhaustion and enhancing psychological well-being across clinical and occupational populations [56], offering a theoretically grounded intervention framework for this substantial subgroup of teachers. The integration of digital tools and teletherapy platforms into cognitive-behavioral interventions offers particular promise for teacher populations, enabling flexible access to evidence-based support without disrupting demanding schedules [57].

Recent theoretical advances underscore that effective professional development for educators experiencing burnout must move beyond simple workload reduction to address deeper cognitive, motivational, and emotional factors [58]. This integrated perspective aligns with the dimensional independence observed in this study, where different burnout components require distinct intervention approaches. 

The Resilient subgroup (32.4%) demonstrates healthy functioning across all dimensions and comprises teachers who could serve as mentors or provide peer support. The Detached subgroup (15.7%) is characterized primarily by low personal accomplishment and moderate depersonalization, and requires interventions to reconnect with educational values and rebuild student relationships through collaborative teaching opportunities. The At-Risk subgroup (2.9%), though small, shows extreme scores across dimensions and requires comprehensive, individualized support, including potential workload reduction, professional counseling, and close monitoring.

#### 4.3.2. Policy Recommendations at the System Level

Policy makers in education should acknowledge the ongoing psychological impact of COVID-19, as nearly 40 percent of educators report being significantly affected by it. Support for recovery should focus on providing emotional resources rather than assuming a uniform experience for professionals in general. This association between high pandemic-related burdens and increased emotional exhaustion indicates that crisis response plans should include emotional support systems, in addition to operational continuity planning.

The protective effect of experience against depersonalization underscores the significance of mentoring programs that pair veteran and novice teachers. Such programs could help veterans learn new strategies for managing stress and provide opportunities to start over by mentoring. Differences in depersonalization between cities and rural areas also indicate that rural teachers require greater support through virtual professional communities, more effective resource allocation, and regular exchanges between urban and rural teachers.

### 4.4. Proposed Multilevel Resilience Framework

**Figure 2:**
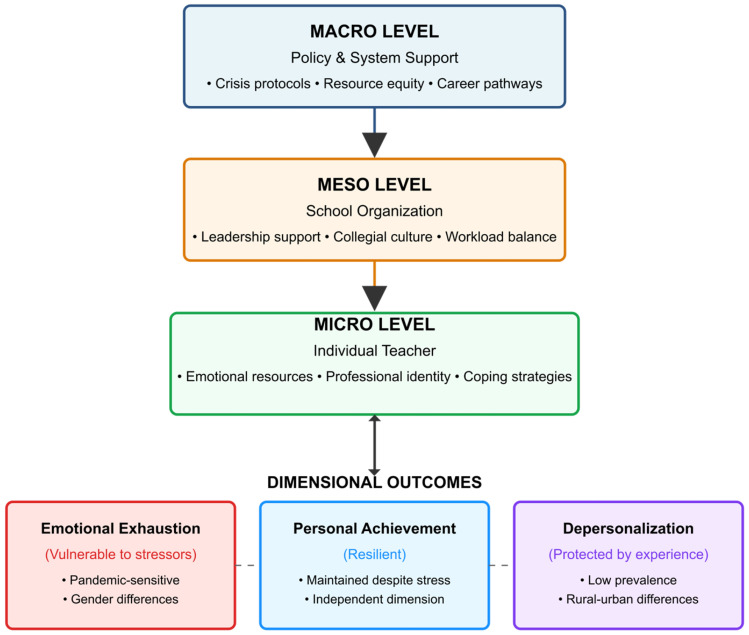
Multilevel Teacher Resilience Framework (MTRF).

Based on our findings, we propose a Multilevel Teacher Resilience Framework (MTRF) that conceptualizes burnout prevention through interconnected intervention levels (Figure 2).

In these two frameworks, it becomes evident that interventions at different levels can have varying impacts across the dimensions of burnout. The micro level focuses on individual resources and coping mechanisms. In particular, there is a focus on emotion regulation because external conditions can readily influence it. At the meso level, mechanisms exist to improve schooling factors and make environments more supportive. The macro level centers on mechanisms that enable the development of teaching careers within broader policy considerations. The blue arrows between various strata indicate that there may also be reciprocal impacts on outcomes. For example, burnout could affect policy.

### 4.5. Theoretical Contributions

This study not only adds to burnout theory in various ways. Firstly, this study provides examples of independence between dimensions in specific cultural settings. Here, arguments against the progression of burnout into a global sequence are presented. In other words, there are additional factors to consider when thinking about burnout. These relate to specific exterior conditions, which, in turn, affect burnout dimensions differently rather than producing similar effects. Finally, there are specific protective mechanisms that function against de-personalization but not against other burnout dimensions.

These results are also informative regarding how burnout looks across cultures. The extent to which individuals maintain their intentions to continue pursuing personal achievements despite feeling emotionally drained or overwhelmed may indicate a Greek cultural value of professional honor or of contributing to society through teaching. From a cross-cultural perspective, burnout profiles designed in individualist cultures originating in Western societies may need to be adapted for cultures that are more collectivistic or honor-focused.

### 4.6. Limitations

Several constraints limit the interpretation of the results. First, the cross-sectional nature of the study shows only associations between variables at a single point in time; therefore, it is impossible to determine whether a cause-and-effect relationship exists. Therefore, it would be advantageous to conduct longitudinal research to investigate the order of events. Second, the self-report instrument is vulnerable to respondent bias, including social desirability (i.e., responding in ways that reflect what respondents believe is socially acceptable), particularly because two of the depersonalization dimensions were considered unacceptable and inappropriate by many respondents. Third, the use of a single-item measure of COVID-19 impact was limited in its ability to efficiently assess the complexity of its impact, as evidenced by the use of comprehensive impact measures. Fourth, because this study evaluated only individuals living within the boundaries of the Achaia region, it is difficult to generalize the findings to other geographic regions of Greece, which have different socioeconomic conditions and/or different resources available to support education. Fifth, there was a large gender difference in the sample, indicating that demographic factors associated with primary education were present; thus, only 8 men participated in the study. Sixth, despite being small in percentage terms, the 9% non-response rate could introduce selection bias if there are differences in burnout among non-participants.

Methodologically, the use of established normal limits may be inadequate for identifying contemporary manifestations of burnout, particularly in the post-pandemic environment. Second, although cluster analysis identified distinct profiles, the study requires an additional validation test to confirm their stability. Finally, without burnout data from sources outside self-reported burnout (e.g., absenteeism due to burnout symptoms, burnout evaluations), the study’s validity cannot be confirmed.

### 4.7. Future Research Directions

The findings of this study suggest several productive avenues for future research. Longitudinal studies tracking teachers from pre-service training through various career stages would illuminate burnout developmental trajectories and clarify whether the protective effect of experience against depersonalization observed here reflects accumulated coping skills or survivor bias. Mixed-methods approaches combining quantitative assessment with in-depth qualitative analysis could elucidate the processes underlying dimensional independence and the cultural mechanisms that appear to protect Greek teachers’ professional efficacy despite emotional strain [6,59,60]. Ecological Momentary Assessment (EMA) methods could capture intraday variation in burnout symptoms and identify real-time precipitating factors, moving beyond the limitations of retrospective global assessments [61,62,63,64,65,66].

The suppression effect identified in this study—whereby COVID-19 burden was associated with depersonalization in bivariate analyses but with emotional exhaustion after controlling for demographics warrants focused methodological investigation. Future studies should examine whether this pattern replicates across educational contexts and cultural settings, and explore demographic variables (particularly gender) that may mediate the pandemic–burnout relationship. Understanding these complex pathways is essential for developing appropriately targeted interventions.

Integration of objective physiological measures with self-report instruments represents a promising methodological advancement. Recent developments in mapping EEG metrics to affective states [67] offer directions for comprehensive burnout assessment protocols that capture both subjective experience and neurophysiological markers. Similarly, biomarkers such as cortisol patterns, inflammatory markers, and sleep quality indicators could provide objective validation of self-reported burnout and reveal physiological mechanisms underlying dimensional differences [68,69,70,71].

Comparative studies across Greek regions and international settings would clarify whether findings reflect culturally specific patterns or universal trends. Such research should prioritize balanced gender representation to enable robust analysis of gender differences; the predominantly female sample in this study (92.2%) limited the interpretation of the significant gender effect on emotional exhaustion. Investigation of whether observed gender differences reflect biological factors, socialized coping styles, or differential exposure to work–family conflict would have important implications for intervention design [72,73].

The differential associations between pandemic burden and burnout dimensions observed here call for a detailed examination of specific pandemic-related stressors. Research should examine whether skills acquired during crisis education—such as technological competencies and adaptive flexibility—provide long-term protective functions against burnout, or whether initial resource investment yields diminishing returns [29,74,75,76]. The remarkable stability of personal accomplishment despite elevated emotional exhaustion merits focused investigation into the cognitive and motivational processes sustaining self-efficacy beliefs under conditions of chronic stress [77,78,79,80,81,82].

Finally, intervention research is needed to test the components of the proposed Multilevel Teacher Resilience Framework (MTRF). Randomized controlled trials comparing dimension-specific interventions (e.g., emotional regulation training for exhaustion, mentoring programs for depersonalization) against generic wellness programs would establish evidence-based best practices. Studies examining the temporal stability of burnout profiles and factors predicting profile transitions would inform early identification and preventive intervention strategies [83,84,85,86,87,88,89,90,91].

## 5. Conclusions

This study of Greek primary school teachers in the post-pandemic period reveals a nuanced picture of occupational burnout characterized by both resilience and vulnerability. The majority of teachers demonstrated low depersonalization and maintained moderate-to-high personal accomplishment, suggesting that Greek educators have developed effective coping mechanisms despite extraordinarily challenging working conditions. However, emotional exhaustion presented a more concerning pattern, with a substantial minority of teachers reporting high levels.

A key methodological contribution of this study is the identification of a suppression effect in the relationship between COVID-19 psychological burden and burnout dimensions. While bivariate analysis showed pandemic burden significantly correlated with depersonalization but not emotional exhaustion, multivariate regression revealed the opposite pattern: controlling for demographic factors, pandemic burden emerged as the strongest predictor of emotional exhaustion while its association with depersonalization became non-significant. This finding underscores the importance of examining both direct and demographically mediated pathways when studying pandemic-related occupational stress, as simple correlational analyses may obscure or misrepresent true relationships.

The dimensional independence observed in this sample—particularly personal achievement’s negligible correlations with emotional exhaustion and depersonalization—challenges the conventional view of burnout as a unified syndrome with predictable sequential progression. Greek teachers appear capable of maintaining professional efficacy and positive student relationships even while experiencing emotional depletion, suggesting that cultural values emphasizing educational vocation and professional honor may serve as protective buffers for specific burnout dimensions.

Experience emerged as a selective protective factor, with years of service and age negatively correlated with depersonalization but not emotional exhaustion. This differential pattern implies that veteran teachers develop interpersonal resilience through accumulated classroom experience, while emotional demands remain constant across career stages. Interventions should therefore target emotional resource restoration for teachers at all experience levels while leveraging mentorship programs to protect early-career educators against cynicism.

The four distinct burnout profiles identified—Emotionally Strained, Resilient, Detached, and At-Risk—provide a framework for differentiated intervention strategies. The largest subgroup requires stress management and workload optimization, while the small At-Risk group necessitates intensive, individualized support. These findings support a multilevel intervention framework addressing individual coping resources, organizational support structures, and systemic policy considerations.

The resilience demonstrated by Greek primary teachers—maintaining professional commitment and interpersonal effectiveness despite emotional challenges—offers grounds for cautious optimism as we move into the post-pandemic era. The cultural emphasis on education’s societal value and teachers’ professional identity appears to provide protective resources that can be leveraged in future intervention efforts. These findings highlight that burnout has multiple causes and manifests differently across dimensions, and that the commitment to professional role performance among Greek teachers, even amid emotional adversity, represents both a source of hope and a resource for overcoming future challenges in creating a better education system.

## Figures and Tables

**Table 1:** Demographic and Professional Characteristics of Participants (*N* = 102).

Characteristic	*n* (%) or M ± SD	Range
Gender		
Female	94 (92.2)	
Male	8 (7.8)	
Age (years)	39.40 ± 11.28	23–59
Educational Qualifications		
Bachelor’s degree only	26 (25.5)	
Master’s degree	56 (54.9)	
Second degree	9 (8.8)	
Doctorate	1 (1.0)	
Other certifications	23 (22.5)	
Years of Service	13.94 ± 11.43	0–35
Marital Status		
Unmarried	45 (44.1)	
Married with children	47 (46.1)	
Married without children	36 (35.3)	
Divorced	4 (3.9)	
School Location		
Urban	83 (81.4)	
Rural/Semi-urban	19 (18.6)	
School Size (departments)	9.88 ± 4.58	0–30
Administrative Role		
Current principal	0 (0.0)	
Principal during pandemic	4 (3.9)	
COVID-19 Psychological Impact		
Not at all	4 (3.9)	
A little	15 (14.7)	
Moderately	34 (33.3)	
Quite a lot	40 (39.2)	
Extremely	9 (8.8)	

Note: Categories are not mutually exclusive; “Married without children” includes those currently married regardless of prior children.

**Table 2:** Burnout Dimensions: Descriptive Statistics and Prevalence Categories (*N* = 102).

Dimension	M ± SD	Median	Skewness	Kurtosis	Low (%)	Moderate (%)	High (%)
Emotional Exhaustion	24.56 ± 13.13	24.50	0.42	−0.78	43 (42.2)	23 (22.5)	36 (35.3)
Personal Achievement	38.90 ± 5.25	39.00	−0.40	−0.29	34 (33.3)	47 (46.1)	21 (20.6)
Depersonalization	4.83 ± 6.02	2.50	2.28	6.58	69 (67.6)	14 (13.7)	19 (18.6)

Note. Emotional Exhaustion: Low ≤ 20, Moderate 21–30, High ≥ 31

**Table 3:** Pearson Correlations Between Burnout Dimensions and Professional Variables.

Variable	Emotional Exhaustion	Personal Achievement	Depersonalization
Age	0.178	0.063	−0.170 *
Years of Service	0.178	0.080	−0.144 *
School Departments	−0.164	−0.112	−0.066

* *p* < 0.05.

**Table 4:** Correlations Between COVID-19 Psychological Burden and Burnout Dimensions.

Burnout Dimension	Pearson r	*p*-Value	R^2^	95% CI	Interpretation
Emotional Exhaustion	0.078	0.435	0.006	[−0.12, 0.27]	Negligible
Personal Achievement	−0.017	0.353	0.009	[−0.29, 0.11]	Negligible
Depersonalization	0.339 ***	0.001	0.115	[0.15, 0.51]	Small Effect

*** *p* < 0.001.

**Table 5:** Intercorrelations Among Burnout Dimensions.

Dimension	1	2	3
1. Emotional Exhaustion	—		
2. Personal Achievement	0.003	—	
3. Depersonalization	0.488 ***	−0.024	—

*** *p* < 0.001.

**Table 6:** Burnout Profiles from Cluster Analysis.

Profile	n (%)	Emotional Exhaustion M (SD)	Personal Achievement M (SD)	Depersonalization M (SD)	Key Characteristics
Resilient	33 (32.4)	11.67 (4.51)	41.91 (3.66)	0.88 (0.89)	Low all dimensions
Emotionally Strained	50 (49.0)	32.76 (9.42)	39.20 (3.12)	5.60 (4.57)	Moderate EE, maintained PA
At-Risk	3 (2.9)	54.00 (0.00)	48.00 (0.00)	4.83 (3.27)	Elevated all dimensions
Detached	16 (15.7)	20.00 (6.51)	30.06 (2.29)	5.88 (3.14)	High DP primarily

Note: PA = Personal Achievement; DP = Depersonalization; EE = Emotional Exhaustion.

**Table 7:** Hierarchical Regression Models Predicting Burnout Dimensions.

Predictor	Emotional Exhaustion	Personal Achievement	Depersonalization
	β (SE)	β (SE)	β (SE)
Step 1: Demographics	R^2^ = 0.089	R^2^ = 0.028	R^2^ = 0.115 *
Gender (Female)	0.21 * (2.84)	−0.05 (1.93)	−0.08 (1.21)
Age	0.14 (0.19)	0.09 (0.13)	−0.18 (0.08)
Step 2: Professional	ΔR^2^ = 0.043	ΔR^2^ = 0.019	ΔR^2^ = 0.087 *
Years of Service	0.08 (0.21)	−0.04 (0.14)	−0.19 * (0.09)
School Location (Rural)	0.09 (1.76)	−0.07 (1.20)	0.22 * (0.75)
Step 3: COVID-19	ΔR^2^ = 0.247 ***	ΔR^2^ = 0.008	ΔR^2^ = 0.015
Pandemic Burden	0.52 *** (1.23)	−0.09 (0.84)	0.13 (0.52)
Total R^2^	0.136 *	0.055	0.225 ***

* *p* < 0.05, *** *p* < 0.001.

## Data Availability

The data presented in this study are available on request from the corresponding author.

## Supplementary Materials

The following supporting information can be downloaded at https://www.mdpi.com/article/10.3390/ijerph23020190/s1. Table S1. Variable Codebook and Scoring Information. Table S2. MBI-ES Item Descriptive Statistics (N = 102). Table S3. Internal Consistency Reliability (Cronbach’s Alpha). Table S4. Normality Assessment for Burnout Dimensions. Table S5. Complete Correlation Matrix (Pearson and Spearman). Table S6. Variance Inflation Factors (VIF) for Regression Predictors. Table S7. Cluster Analysis Validation Metrics. Text S1. Scoring Formula Reference.
