# Supplementary material for: Teacher Well-Being and Burnout Resilience: Dimensional Independence, Pandemic Burden, and Profile Analysis in Primary Education
Source: Int J Environ Res Public Health. 2026 Jan 31;23(2):190. doi: 10.3390/ijerph23020190 (PMC12940852; doi:10.3390/ijerph23020190)
Supplement: Supplementary file 1 [file ijerph-23-00190-s001.zip › ijerph-4053438-supplementary.pdf]

Article

Teacher Well-Being and Burnout Resilience: Dimensional Independence, Pandemic Burden, and Profile Analysis in Primary Education

Supplementary material

Table S1. Variable Codebook and Scoring Information.

A. Demographic Variables

Variable (Greek)	Variable (English)	Coding	Range
Ηλικία	Age	Continuous (years)	23-59
Φύλο	Gender	1=Male, 2=Female	1-2
Gender_Female	Gender (recoded)	0=Male, 1=Female	0-1
Πρόσθετες_σπουδές	Education	1=Doctorate, 2=Master's, 3=Second degree, 4=SELDE, 5=Further training, 6=Bachelor's only	1-6
Οικογενειακή_κατάσταση	Marital Status	1=Unmarried, 2=Married no children, 3=Married with children, 4=Divorced	1-4
Περιοχή_έδρας_σχολείου	School Location	1=Urban, 2=Rural/Semi-urban	1-2
Location_Rural	Location (recoded)	0=Urban, 1=Rural	0-1
Αριθμός_τμημάτων	School Size	Number of departments	0-30
Έτη_υπηρεσίας	Years of Service	Continuous (years)	0-35
Πανδημία_Covid	COVID-19 Impact	1=Not at all to 5=Extremely	1-5

B. MBI-ES Item Assignments

Dimension	Items	N Items	Score Range
Emotional Exhaustion (EE)	Q1-Q9 (Ερώτηση1-9)	9	0-54
Personal Achievement (PA)	Q10-Q17 (Ερώτηση10-17)	8	0-48
Depersonalization (DP)	Q18-Q22 (Ερώτηση18-22)	5	0-30

Note: All items scored on 0-6 Likert scale (0=Never, 1=A few times a year, 2=Once a month, 3=A few times a month, 4=Once a week, 5=A few times a week, 6=Every day)

C. Greek MBI-ES Cutoff Values

Dimension	Low	Moderate	High
Emotional Exhaustion	≤20	21-30	≥31
Depersonalization	≤5	6-10	≥11
Personal Achievement	≤35 (burnout)	36-41	≥42 (healthy)

Reference: Anagnostopoulos, F.; Papadatou, D. Factorial composition and internal consistency of the Greek version of the Maslach Burnout Inventory. *Psychologika Themata* **1992**, *5*, 183–202. (In Greek)

Table S2. MBI-ES Item Descriptive Statistics (N = 102)

Emotional Exhaustion Items (Q1-Q9)

Item	M	SD	Min	Max
Q1	3.39	1.55	1	6
Q2	3.02	1.69	0	6
Q3	2.20	1.86	0	6
Q4	3.03	1.81	0	6
Q5	2.27	1.89	0	6
Q6	4.15	1.78	0	6
Q7	2.20	1.80	0	6
Q8	2.14	1.71	0	6
Q9	2.17	2.09	0	6

Personal Achievement Items (Q10-Q17)

Item	M	SD	Min	Max
Q10	5.21	0.88	3	6
Q11	4.81	0.96	2	6
Q12	4.99	1.19	1	6
Q13	4.48	1.15	1	6
Q14	5.24	0.53	4	6
Q15	4.70	1.23	1	6
Q16	4.65	1.10	2	6
Q17	4.83	1.18	2	6

Depersonalization Items (Q18-Q22)

Item	M	SD	Min	Max
Q18	1.02	1.75	0	6
Q19	0.97	1.49	0	6
Q20	1.01	1.51	0	6
Q21	1.31	1.45	0	6
Q22	0.52	1.25	0	6

Table S3. Internal Consistency Reliability (Cronbach's Alpha)

Dimension	α	N Items	Interpretation
Emotional Exhaustion	0.934	9	Excellent
Personal Achievement	0.778	8	Good
Depersonalization	0.863	5	Good

Note: $\alpha > 0.90$ = Excellent; 0.80-0.89 = Good; 0.70-0.79 = Acceptable

Table S4. Normality Assessment for Burnout Dimensions

Dimension	Skewness	Kurtosis	K-S Statistic	K-S p-value	Distribution
Emotional Exhaustion	0.42	-0.78	0.120	0.0993	Approximately normal
Personal Achievement	-0.40	-0.29	0.125	0.0752	Approximately normal
Depersonalization	2.28	6.58	0.211	0.0002	Positive skew (floor effect)

Note: Kolmogorov-Smirnov test with $p < 0.05$ indicates significant departure from normality. Depersonalization shows floor effects requiring non-parametric analyses.

Table S5. Complete Correlation Matrix (Pearson and Spearman)

Pearson Correlations (r)

Variable	1	2	3	4	5	6
1. EE	—					
2. PA	0.003	—				
3. DP	0.488***	0.010	—			
4. COVID	0.078	-0.017	0.339**	—		
5. Age	0.173	0.063	-0.170	-0.333	—	
6. Years	0.178	0.080	-0.144	-0.374	0.889***	—

Spearman Correlations (ϱ)

Variable	1	2	3	4
1. EE	—			
2. PA	-0.105	—		
3. DP	0.392***	-0.327	—	
4. COVID	0.081	-0.037	0.300**	—

Note: *$p < 0.05$, **$p < 0.01$, ***$p < 0.001$

Table S6. Variance Inflation Factors (VIF) for Regression Predictors

Predictor	VIF	Tolerance	Interpretation
Gender (Female)	1.14	0.877	No multicollinearity
Age	5.05	0.198	Moderate (expected with Years)
Years of Service	5.24	0.191	Moderate (expected with Age)
Location (Rural)	1.25	0.800	No multicollinearity
COVID-19 Burden	1.26	0.794	No multicollinearity

Note: VIF < 5 indicates acceptable multicollinearity. Age-Years collinearity (r = 0.89) is developmentally expected and does not invalidate analyses when both are included.

Table S7. Cluster Analysis Validation Metrics

Determining Optimal Number of Clusters

N Clusters	Agglomeration Coefficient	Coefficient Change	Calinski-Harabasz
2	89.45	—	45.2
3	67.21	22.24	52.8
4	52.48	14.73	63.4
5	43.12	9.36	58.1

Decision: 4-cluster solution selected based on elbow method (largest coefficient change at 3→4) and Calinski-Harabasz index maximization.

Cross-Validation Accuracy

Method	Classification Accuracy
Discriminant Function Analysis	95.1%
Leave-One-Out Cross-Validation	89.2%
Bootstrap Jaccard Stability (mean)	0.76

Text S1. Scoring Formula Reference

Computing Burnout Dimension Scores

Emotional Exhaustion (EE_Sum) = Q1 + Q2 + Q3 + Q4 + Q5 + Q6 + Q7 + Q8 + Q9

Personal Achievement (PA_Sum) = Q10 + Q11 + Q12 + Q13 + Q14 + Q15 + Q16 + Q17

Depersonalization (DP_Sum) = Q18 + Q19 + Q20 + Q21 + Q22

Category Assignment

EE_Category:

IF EE_Sum ≤ 20 THEN 1 (Low)

IF EE_Sum 21-30 THEN 2 (Moderate)

IF EE_Sum ≥ 31 THEN 3 (High)

DP_Category:

IF DP_Sum ≤ 5 THEN 1 (Low)

IF DP_Sum 6-10 THEN 2 (Moderate)

IF DP_Sum ≥ 11 THEN 3 (High)

PA_Category:

IF PA_Sum ≤ 35 THEN 1 (Low = Burnout)

IF PA_Sum 36-41 THEN 2 (Moderate)

IF PA_Sum ≥ 42 THEN 3 (High = Healthy)
